# Letter to the editor: “Utilization of CT and MRI scanning in Taiwan, 2000–2017”

**DOI:** 10.1186/s13244-023-01420-x

**Published:** 2023-05-15

**Authors:** Hao-Ming Li, Shi-Zuo Liu, Lee-Ren Yeh, Nan-Han Lu, Liang-Yi Wang

**Affiliations:** 1 Department of Public Health, College of Medicine, National Cheng Kung University, No. 138, Sheng Li Road, Tainan City, 704 Taiwan; 2 Department of Medical Imaging, E-Da Hospital, I-Shou University, No. 1, Yida Road, Yanchao District, Kaohsiung City, 824 Taiwan; 3 Department of Medical Imaging and Radiological Sciences, College of Medicine, I-Shou University, No. 8, Yida Road, Yanchao District, Kaohsiung City, 824 Taiwan; 4 School of Medicine, College of Medicine, I-Shou University, No. 8, Yida Road, Yanchao District, Kaohsiung City, 824 Taiwan; 5 Department of Pharmacy, Tajen University, No. 20, Weixin Rd., Yanpu Township, Pingtung County, 907 Taiwan

Dear Editor in Chief,

We read with interest the article entitled “Utilization of CT and MRI scanning in Taiwan, 2000–2017” by Huang et al. [1] recently published in *Insights into Imaging*. The authors conducted comprehensive analyses of the advanced imaging utilization in Taiwan across subgroups, using national data from National Health Insurance (NHI) claims and Organisation for Economic Co-operation and Development (OECD) health statistics. We congratulate the authors for their contributions to provide an overview of overall advanced imaging examinations performed in Taiwan. However, our examination of the study has revealed several limitations in the authors' approach to subgroup analysis.

First, using the entire population as the denominator of utilization rates in subgroups would cause an underestimation of the true rates. For instance, in the emergency department (ED) setting, the reported rates of CT use in Taiwan ranged from 116 to 177 per 1000 ED visits during 2005–2013 [2–4], compared to rates reported in other countries ranging from 34 to 245 per 1000 ED visits (as summarized in Table 1) [5–11]. However, the rate reported by the authors was 16 per 1000 persons per year, calculated by using the whole population of Taiwan (approximately 24 million) as the denominator. This could lead to confusion that Taiwan had a low CT utilization rate in ED setting compared to other countries. The appropriate denominator should be the yearly users of ED services (approximately 3–4 million). The same issue applies to rates reported in other subgroups (as shown in Table 4 of the authors’ article) [1].

**Table 1:** Worldwide trends of CT use in the ED patients

Country	Setting	Study	Rates per 1000 ED visits			References
		Periods	2005	2009	2013	
Taiwan	Single center	2005–2020	116	111–154	177	[2–4]
US	National	2004–2016	107–153	211	245	[5, 6]
Canada	Regional	2003–2009	55	82	NR	[7, 8]
Australia	Regional	2003–2015	61	64	82	[9]
Korea	Single center	2001–2010	220	224	NR	[10]
Iceland	Single center	2002–2017	34	59	87	[11]

NR, not reported

Second, the underestimation of CT scans due to under-coding and self-pay should not be ignored. Previous studies have shown the underreporting of CT and MRI in US inpatient claims data [12], which may also occur in Taiwan. The NHI Bureau limits the reimbursement of multiple CT scans for a patient at the same day and imposes penalties for outliers in service volumes, leading to changes in provider behavior such as discretionary billing [13]. To observe this phenomenon, we compared the number of CT scans in claim records with total CT scans, including uncoded and self-pay scans, in our hospital during 2010–2017. The underestimation of CT scans in claim records was up to 10.2% in emergency patients and 8.8% in outpatients in 2017 (as shown in Table 2). We also found increasing trends of uncoded CT scans in emergency patients and self-pay CT scans in outpatients. These findings suggest that the actual rates of CT utilization may be higher than those derived from NHI claims data, and the difference may continue to increase in the future.

**Table 2:** Trends of all CTs, uncoded and self-paid CT during 2010–2017, stratified by patient settings

Setting	2010	2011	2013	2015	2017	Trend
*Emergency*						
All CTs	8694	10,073	10,401	12,654	12,317	*
Rate (per 1000)^a^	125	142	156	185	199	*
Uncoded (*n*, %)^b^	373 (4)	519 (5)	688 (7)	1022 (8)	1234 (10)	*
Self-pay (*n*, %)^b^	11 (0)	9 (0)	5 (0)	9 (0)	23 (0)	
Difference (%)^c^	4.4	5.2	6.7	8.1	10.2	*
*Inpatients*						
All CTs	3963	4223	3871	3824	3060	#
Rate (per 1000)^a^	90	96	90	83	76	#
Uncoded (*n*, %)^b^	45 (1)	38 (1)	50 (1)	50 (1)	48 (2)	*
Self-pay (*n*, %)^b^	5 (0)	3 (0)	8 (0)	14 (0)	7 (0)	
Difference (%)^c^	1.3	1	1.5	1.7	1.8	*
*Outpatients*						
All CTs	11,319	12,179	13,626	15,168	14,039	*
Rate (per 1000)^a^	26	28	32	34	37	*
Uncoded (*n*, %)^b^	3 (0)	2 (0)	5 (0)	1 (0)	11 (0)	
Self-pay (*n*, %)^b^	351 (3)	457 (4)	614 (5)	917 (6)	1219 (9)	*
Difference (%)^c^	3.1	3.8	4.5	6.1	8.8	*

*Significant increasing trend

^#^Significant decreasing trend

^a^The denominator of rates were the number of patient visits in the corresponding setting

^b^The denominator of percentages were the number of all CTs in the corresponding setting

^c^The calculation of difference was the number of CTs not in claim records divided by the number of all CTs in the corresponding setting

Finally, the use of OECD health statistics data to compare advanced imaging utilization across countries is limited. Although the data showed in the hospital and ambulatory subgroups primarily refer to inpatient and outpatient settings, exceptions are described in the Definitions, Sources and Methods of OECD health statistics [14]. As the countries selected in eTable 4 of the authors’ article [1], the data of “in-hospital” CT examinations in Korea and the USA include outpatients, while the data of “ambulatory” CT examinations in France, Korea and the USA include inpatients. In addition to the mixed data between inpatient and outpatient settings, the methodology and coverage of data source vary widely across countries in OECD health statistics. Given these limitations, using data from NHI claims and OECD health statistics may not be suitable for the subgroup analyses of the imaging utilization.

In conclusion, our review of the article by Huang et al. on the utilization of CT and MRI scanning in Taiwan highlights several limitations in the subgroup analyses. Using the entire population as the denominator in calculating utilization rates has led to underestimation of the actual rates. Additionally, the underreporting of CT and MRI scans due to under-coding and self-pay is not negligible and may have resulted in further underestimation. The limitations of using data from NHI claims and OECD health statistics for subgroup analyses of advanced imaging utilization also warrant attention. It is recommended that future studies on advanced imaging utilization in Taiwan consider data from multiple hospitals to mitigate the impact of under-coding and self-pay, and to accurately reflect the utilization rates in both inpatient and outpatient settings.

## Data Availability

All data and material are included in this Letter to the Editor.

## Abbreviations

CT: Computed tomography
ED: Emergency Department
MRI: Magnetic resonance imaging
NHI: National Health Insurance
OECD: Organisation for Economics Co-operation and Development
